# AMPK-NF-κB Axis in the Photoreceptor Disorder during Retinal Inflammation

**DOI:** 10.1371/journal.pone.0103013

**Published:** 2014-07-21

**Authors:** Mamoru Kamoshita, Yoko Ozawa, Shunsuke Kubota, Seiji Miyake, Chiduru Tsuda, Norihiro Nagai, Kenya Yuki, Shigeto Shimmura, Kazuo Umezawa, Kazuo Tsubota

**Affiliations:** 1 Laboratory of Retinal Cell Biology, Keio University School of Medicine, Shinjuku, Tokyo, Japan; 2 Department of Ophthalmology, Keio University School of Medicine, Shinjuku, Tokyo, Japan; 3 Department of Molecular Target Medicine Screening, Aichi Medical University School of Medicine, Nagakute, Aichi, Japan; University Zürich, Switzerland

## Abstract

Recent progress in molecular analysis has revealed the possible involvement of multiple inflammatory signaling pathways in pathogenesis of retinal degeneration. However, how aberrant signaling pathways cause tissue damage and dysfunction is still being elucidated. Here, we focus on 5′-adenosine monophosphate (AMP)-activated protein kinase (AMPK), originally recognized as a key regulator of energy homeostasis. AMPK is also modulated in response to inflammatory signals, although its functions in inflamed tissue are obscure. We investigated the role of activated AMPK in the retinal neural damage and visual function impairment caused by inflammation. For this purpose, we used a mouse model of lipopolysaccharide-induced inflammation in the retina, and examined the effects of an AMPK activator, 5-aminoimidazole-4-carboxamide ribonucleoside (AICAR). During inflammation, activated AMPK in the neural retina was decreased, but AICAR treatment prevented this change. Moreover, the electroretinogram (ERG) a-wave response, representing photoreceptor function, showed visual dysfunction in this model that was prevented by AICAR. Consistently, the model showed shortened photoreceptor outer segments (OSs) with reduced levels of rhodopsin, a visual pigment concentrated in the OSs, in a post-transcriptional manner, and these effects were also prevented by AICAR. In parallel, the level of activated NF-κB increased in the retina during inflammation, and this increase was suppressed by AICAR. Treatment with an NF-κB inhibitor, dehydroxymethylepoxyquinomicin (DHMEQ) preserved the rhodopsin level during inflammation, suppressing NF-κB. These findings indicated that AMPK activation by AICAR and subsequent NF-κB inhibition had a protective effect on visual function, and that AMPK activation played a neuroprotective role during retinal inflammation.

## Introduction

Recent progress in the elucidation of disease mechanisms has led to the discovery of changes in multiple inflammatory signaling pathways that are pathogenic; This is also the truth in the field of the retinal diseases. Some of these pathways have been investigated in detail, leading to molecular targeting therapies. For example, anti-vascular endothelial growth factor (anti-VEGF) is used clinically to inhibit vascular proliferation and permeability in age-related macular degeneration [1] and diabetic retinopathy [2]. However, many of the molecular mechanisms that underlie pathological disorders in the neural retina remain to be elucidated.

Here, we focused on the role of 5′-adenosine monophosphate (AMP)-activated protein kinase (AMPK) in the pathogenesis of retinal inflammation. AMPK is composed of three subunits: an α catalytic subunit, and β and γ regulatory subunits. The β subunit has a glycogen-binding domain and a tethering domain for the α and γ subunits, and the γ subunit has the binding site that enables AMP to activate the complex. Each subunit has isoforms, all of which are encoded by distinct genes: α1 and α2, β1 and β2, and γ1, γ2, and γ3. The enzyme is activated when the Thr 172 residue of the α subunit is phosphorylated by an upstream kinase, such as tumor suppressor LKB1 [3], Ca^2+^/calmodulin-dependent protein kinase kinase (CaMKK) [4], or transforming growth factor-β-activated kinase (TAK1) [5], [6].

AMPK is well known for its role in cellular energy homeostasis. An increase in the AMP/ATP ratio by starvation activates AMPK to phosphorylate key target proteins that induce energy production from glucose and fatty acids, and that inhibit energy consumption by protein, cholesterol, and glycogen synthesis [6]–[9]. Also, AMPK can be activated by exercise through depletion of ATP [10]. Decreased AMPK activity is observed in obesity [11], in which it effectively decreases fatty acid metabolism and increases insulin resistance [12]. AMPK is also involved in several housekeeping processes, including endoplasmic reticulum stress, oxidative stress responses, apoptosis, and autophagy [10]. AMPK-deficient mice show increased endoplasmic reticulum stress, leading to arteriosclerosis [13], while activated AMPK can attenuate the damage in vascular endothelial cells under oxidative stress by modifying the bioactivity of nitric oxide (NO) and the level of reactive oxygen species (ROS) [14]. Some hormones, such as adiponectin, ghrelin, and leptin, can either activate or inhibit AMPK signaling in a tissue-specific manner [10], [15]. All of these actions are associated with cellular responses to changes in the microenvironment. Downstream of AMPK, various signaling cascades are modulated, via SIRT1, FoxO, p53, mTOR, NF-κB, and others [10]. Because imbalance of cellular response could cause tissue damage and dysfunction, we hypothesized that the changes in AMPK activation may be involved in the disease pathogenesis.

In animal experiments, 5-aminoimidazole-4-carboxamide ribonucleoside (AICAR) is widely used to activate AMPK. AICAR is an adenosine analog that is taken into cells through adenosine transporters, phosphorylated, and converted to ZMP, which binds to the γ subunit of AMPK to activate the enzyme [16], [17].

Retinal diseases involving inflammation of the neural retina can severely degrade visual function [18]. One animal model used to analyze retinal inflammation is the mouse endotoxin-induced retinitis and uveitis (EIU) model, in which lipopolysaccharide (LPS) is the endotoxin [19]. In the retina of this model, inflammatory factors, such as interleukin-6 (IL-6) and angiotensin II, are released [20]–[23], and the downstream intracellular inflammatory signaling pathways as well as ROS are induced [24], to promote visual function impairment that is recorded by electroretinogram (ERG) and reduction of the level of the visual pigment, rhodopsin [21], [23]–[25]. Recruitment of inflammatory cells and intraocular leakage of proteins including cytokines are also reported in this model that, at least in part, involves relationship to AMPK activation [26]. However, the cellular response in the neural retina, in terms of AMPK involvement, remains to be elucidated.

In this study, we analyzed the level of activated AMPK and its influence on visual function in the inflamed retina, together with AICAR’s effects, using the mouse EIU model. Given that AMPK may alter various kinds of signaling pathways, changes in the level of activated AMPK may contribute to their imbalance and cellular dysfunction which can lead to visual function impairment during inflammation. We focused on one type of retinal neuronal cells, the rod photoreceptor cells, which represent the largest cell population in the retina, and convert light stimuli to the electric signal that is transmitted to subsequent neurons to create vision. Furthermore, in order to evaluate whether NF-κB is involved in the inflammatory disease pathogenesis downstream of AMPK, we used a specific inhibitor of NF-κB, dehydroxymethylepoxyquinomicin (DHMEQ).

## Materials and Methods

### Ethics Statement

All animal experiments were conducted in accordance with the Association for Research in Vision and Ophthalmology (ARVO) Statement for the Use of Animals in Ophthalmic and Vision Research, the ARRIVE (Animal Research: Reporting *In Vivo* Experiments) guidelines, and the guideline for the ethics committee of Keio University. The protocol for this study was approved by the Keio University Institutional Animal Care and Use Committee (permission No. 08002) (Tokyo, Japan).

### Animals and treatments

Six-week-old male C57BL/6 mice were purchased (Clea Japan, Tokyo, Japan) and maintained in an air-conditioned room under a 12-h dark/light cycle, with free access to food and water. Mice received a single intraperitoneal injection of 6.0 mg/kg body weight (BW) LPS from *Escherichia coli* (Sigma-Aldrich, St. Louis, MO, USA) in phosphate-buffered saline (PBS) to generate the EIU model. PBS was injected for the control. Mice were treated with the specific AMPK activator, AICAR (Santa Cruz Biotechnology, Santa Cruz, CA, USA), at 250 mg/kg BW or PBS as vehicle, 3 hours before the LPS injection. Alternatively, mice were intraperitoneally injected with an NF-κB inhibitor (DHMEQ), which inhibits the nuclear translocation of NF-κB and its DNA binding without affecting the phosphorylation or degradation of IκB-α [27]–[29], at 10 mg/kg BW or with PBS containing 4% dimethyl sulfoxide (DMSO) as the vehicle, 2 hours before the LPS injection. For sampling the retina, animals were anesthetized with 60 mg/kg BW of pentobarbital sodium (Dainippon Sumitomo Pharmaceutical Co., Osaka, Japan) intraperitoneal injection and sacrificed by cervical dislocation.

### Immunoblot Analyses

The eyes were enucleated, and each retina was isolated and placed in lysis buffer, including protease inhibitor cocktail (Complete, EDTA-free; Roche, Mannheim, Germany) and phosphatase inhibitor cocktails 2 and 3 (Sigma-Aldrich), to prepare the lysate. Then, the lysate was treated with Laemmli’s sample buffer and separated by 10% SDS-polyacrylamide gel electrophoresis. The proteins were electrically transferred to a polyvinylidene fluoride membrane (Immobilon-P; Millipore, Bedford, MA, USA) in a Trans-Blot SD Cell (Bio-Rad Laboratories, Hercules, CA, USA). After the transfer, the membrane was blocked with 5% skim milk in TBS-T or TNB blocking buffer (0.1 M Tris-HCl, pH 7.5, 0.15 M NaCl, 0.5% TSA Blocking Reagent [PerkinElmer Life Sciences, Waltham, MA, USA]), then incubated overnight at 4°C with a rabbit anti-rhodopsin polyclonal antibody (1∶100,000; LSL, Osaka, Japan), rabbit anti-AMPKα (1∶200; Cell Signaling Technology, Beverly MA, USA), rabbit anti-phospho-AMPKα (1∶1000; Cell Signaling Technology), rabbit anti-phospho-NF-κB p65 (Ser536) (93H1) antibody (1∶1000; Cell Signaling Technology), or mouse anti-α-tubulin (1∶100,000; Sigma-Aldrich).

The membrane was then incubated with a horseradish peroxidase-conjugated secondary antibody. Finally, the signals were detected using the enhanced chemiluminescence system (ECL Blotting Analysis System; Amersham, Arlington Heights, IL, USA), measured with the ImageJ program (developed by Wayne Rasband, National Institutes of Health, Bethesda, MD; available at http://rsb.info.nih.gov/ij/index.html), and normalized to α-tubulin. The level of rhodopsin protein was measured 24 hours after LPS injection when the reduction of rhodopsin protein is significant in the retina during inflammation according to the previous reports [21], [23]–[25]. Because the levels of rhodopsin monomer and dimer change in parallel in this model [25], which was also confirmed in this study (data not shown), we showed the level of rhodopsin monomer. For comparing the levels of p-AMPK and t-AMPK, we transferred the proteins in different membranes to measure the levels of p-AMPK and t-AMPK, separately, after normalized to α-tubulin in each membrane (data not shown). The levels of p-AMPK and t-AMPK were compared 1.5 hours after LPS injection when p-AMPK level was at the lowest in the retina during inflammation (Fig. S1). The level of activated NF-κB was also compared 1.5 hours after LPS injection, at the same time point as p-AMPK, to discuss the relationship between the levels of p-AMPK and NF-κB.

### Immunohistochemistry

Retinal sections (7-µm thick) were fixed in 4% paraformaldehyde, treated with methanol and acetone mixture for 10 minutes at −20°C, and incubated overnight at 4°C with rabbit anti-phospho-AMPKα antibody (1∶50; Cell Signaling Technology), followed by biotinylated anti-rabbit IgG (1∶200; Vector Laboratories, Burlingame, CA, USA), avidin biotin complex (Vector Laboratories), and fluorescence-conjugated tyramide (PerkinElmer Inc., Waltham, MA, USA). For negative control, blocking solution was applied instead of the primary antibody, and also confirmed using a non-specific rabbit IgG (Cell Signaling Technology) (data not shown). The sections were prepared 1.5 hours after LPS injection when p-AMPK level was at the lowest in the retina during inflammation (Fig. S1). For detecting rhodopsin, fixed sections were incubated overnight at 4°C with mouse anti-rhodopsin antibody (1∶10,000; Thermo Fisher Scientific Inc.) followed by Alexa 488-conjugated goat anti-mouse IgG (Invitrogen, Carlsbad, CA, USA) at room temperature for 1 hour. Nuclei were counterstained with bisbenzamide (1∶1000) from a stock solution of 10 mg/ml (Hoechst 33258; Sigma-Aldrich). The sections were prepared 24 hours after LPS injection when the reduction of rhodopsin protein is significant in the retina during inflammation according to the previous reports [21], [23]–[25]. All the sections were examined under a microscope equipped with a digital camera (Olympus, Co., Tokyo, Japan). Outer segment (OS) lengths were measured in the posterior retina at four points that were 200 and 500 µm apart, two on either side of the optic nerve, using the ImageJ program, and averaged.

### Electroretinogram

Mice were dark-adapted for at least 12 hours and prepared under dim red illumination. They were anesthetized with 60 mg/kg BW of pentobarbital sodium (Dainippon Sumitomo Pharmaceutical Co.) and kept on a heating pad throughout the experiment. The pupils were dilated with one drop of a mixture of 0.5% tropicamide and 0.5% phenylephrine (Santen Pharmaceutical Co., Osaka, Japan). The ground electrode was placed on the tail, and the reference electrode was placed in the mouth. The active electrodes were gold wires placed on the cornea. Recordings were made with a PowerLab System 2/25 (AD Instruments, New South Wales, Australia). Full-field scotopic ERGs were measured in response to flash at intensities ranging from −2.12 to 2.89 log cds/m^2^. Responses were differentially amplified and filtered through a digital bandpass filter ranging from 0.3 to 1000 Hz. Each stimulus was delivered through a commercial stimulator (Ganzfeld System SG-2002; LKC Technologies, Inc., Gaithersburg, MD, USA). The implicit times of the a- and b-waves were measured from the onset of the stimulus to the peak of each wave. The amplitude of the a-wave was measured from the baseline to the trough of the a-wave, and the amplitude of the b-wave was measured from the trough of the a-wave to the peak of the b-wave. The peak points were automatically pointed by the system and confirmed by us. The data were obtained 24 hours after LPS injection when the ERG change is significant in the retina during inflammation according to the previous reports [21], [23]–[25].

### Real-Time RT-PCR

Total RNA was isolated from the retina with TRIzole (Invitrogen) and reverse-transcribed using the High Capacity RNA-to-cDNA Master Mix (Applied Biosystems, Foster City, CA, USA), according to the manufacturer’s instructions. The mRNA levels of rhodopsin were normalized to that of the gapdh mRNA. β-actin and 36B4 mRNAs were also analyzed as normalizers to confirm the data. The forward and reverse primer sequences of rhodopsin were 5′-AACTTCGGCCCCATCTTCA-3′ and 5′-CAGTGGATTCTTGCCGCAG-3′, GAPDH were 5′-AACTTCGGCCCCATCTTCA-3′ and 5′-GATGACCCTTTTGGCTCCAC-3′, β-actin were 5′-AGGTCATCACTATTGGCAACGA-3′ and 5′-GTTTCATGGATGCCACAGGA-3′, and 36B4 were 5′-CGACCTGGAAGTCCAACTAC-3′ and 5′-ATCTGCTGCATCTGCTTG-3′. Real-time PCR was performed using the StepOnePlus™ Real Time PCR system (Applied Biosystems), and the gene expression was quantified using the delta delta CT method. The mRNA was prepared 24 hours after LPS injection when the rhodopsin protein was measured, to compare the mRNA and protein levels at the same time point.

### Statistical analyses

All results are expressed as the mean ± SD. One way ANOVA with Tukey’s post hoc test was used to assess the statistical significance of differences. *P*<0.05 was regarded as significant.

## Results

### Decreased level of activated AMPK in the retina during inflammation and its preservation by AICAR

**Figure 1:**
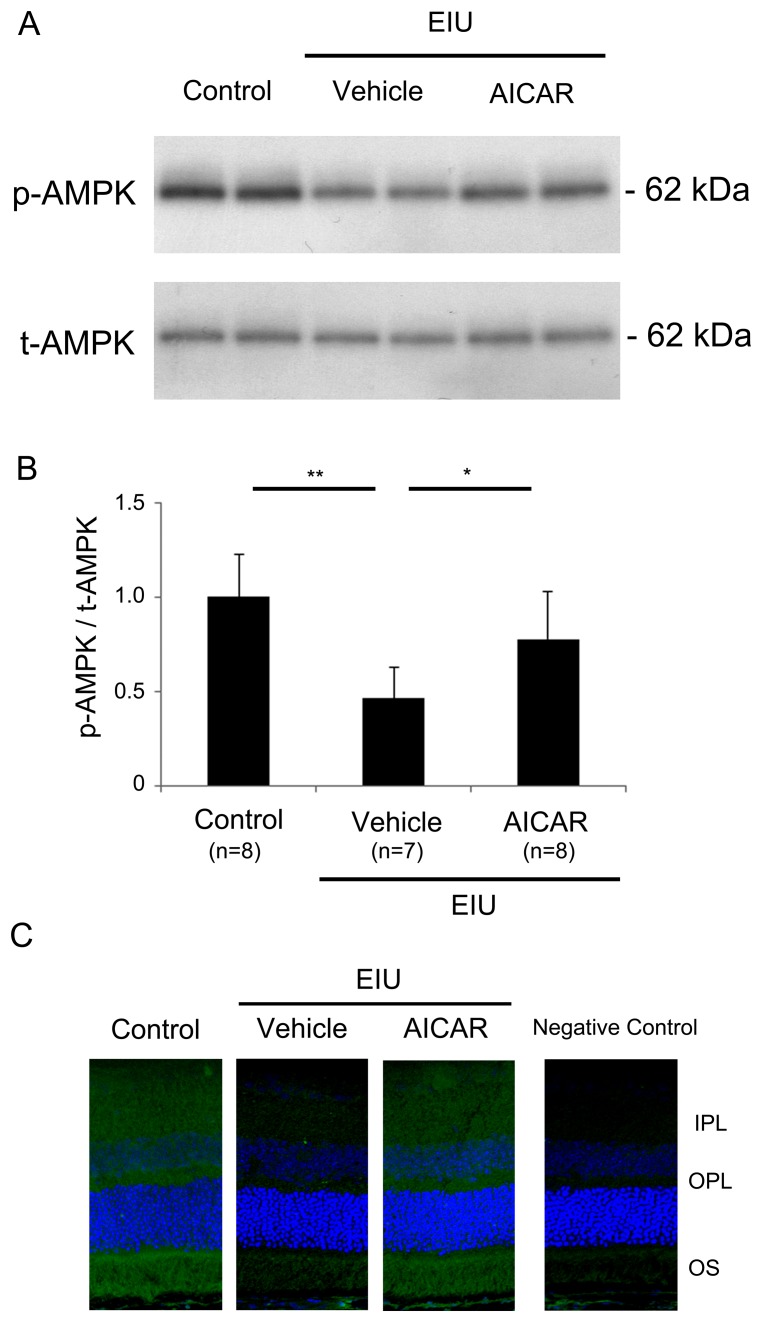
Decrease in activated AMPK in the neural retina during inflammation and its prevention by AICAR. (A, B) Immunoblot analyses. The ratio of p-AMPK/t-AMPK was significantly lower in the retina of vehicle-treated EIU mice than controls, 1.5 hours after LPS injection. AICAR administration significantly increased the ratio during EIU. Both p-AMPK and t-AMPK were measured after normalized to α-tubulin. (C) Immunohistochemistry of p-AMPK. Under control condition, p-AMPK was expressed throughout the retina and retinal pigment epithelium, but the staining was sparse in the retina of vehicle-treated EIU mice, 1.5 hours after LPS injection. The retina of AICAR-treated EIU mice showed clear expression of p-AMPK at the same time point. A negative control staining in the retina of a control animal was also shown. (A, B) *P<0.05. **P<0.01. Control, n = 8; EIU with vehicle treatment, n = 7; EIU with AICAR treatment, n = 8. p-AMPK, phosphorylated AMPK (activated form); t-AMPK, total AMPK; IPL, inner plexiform layer; OPL, outer plexiform layer; OS, outer segment.

To analyze the level of activated AMPK, we measured both the phosphorylated (activated) form of AMPK and the total AMPK in the retina, by immunoblot analysis (Fig. 1A). The ratio of activated to total AMPK in the retina 1.5 hours after LPS injection was reduced when treated with vehicle, compared to the control without inflammation (P<0.01) (Fig. 1B). However, treatment with the AMPK activator, AICAR attenuated the reduction in activated AMPK during inflammation (P<0.05). Total AMPK normalized to α-tubulin was not changed both under control condition and during inflammation with or without AICAR treatment (data not shown).

Immunohistochemistry also showed that staining of the phosphorylated and activated AMPK spread throughout the neural retina under control condition was sparsely observed 1.5 hours after LPS injection when treated with vehicle. However, the staining was preserved in the retina of AICAR treated mice after LPS injection, consistent with the result of immunoblot analysis (Fig. 1C).

### Protective effect of AICAR on visual function during inflammation

**Figure 2:**
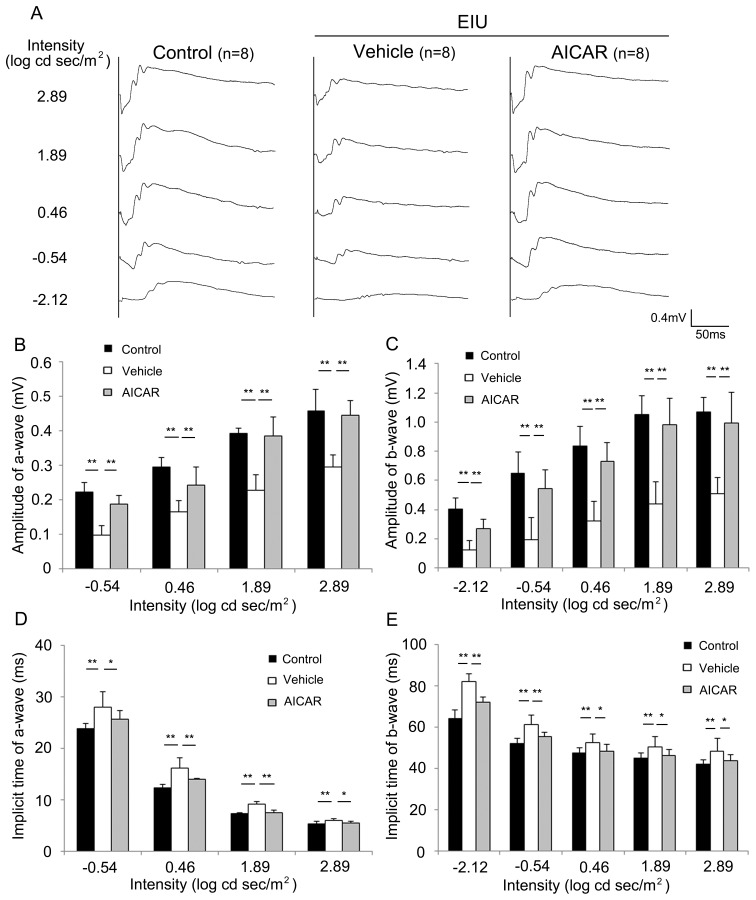
Protective effect of AICAR on visual function during inflammation. (A–E) ERG data 24 hours after LPS injection. (A) Representative wave responses of scotopic ERG intensity series from an individual mouse. The amplitudes of the a-wave (B) and b-wave (C) were decreased during EIU, but AICAR treatment clearly prevented the decrease. The implicit times of the a-wave (D) and b-wave (E) were prolonged during EIU, but this effect was significantly avoided by AICAR. *P<0.05. **P<0.01. All groups, n = 8.

Next, in order to examine the effects of the reduced level of activated AMPK and of AICAR treatment on visual function during inflammation, we performed ERGs (Fig. 2A–E). In the ERG, the a-wave reflects photoreceptor cell function, and the b-wave reflects the electrical activity in the inner retina, which is stimulated after the photoreceptor. ERGs recorded 24 hours after the LPS injection showed a reduction in the a-wave and b-wave amplitudes compared with the control, and the reductions were prevented by AICAR treatment (P<0.01) (Fig. 2A, B, C). Consistent with this result, the implicit times of the a-wave and b-wave were prolonged during inflammation, and the influence was also suppressed by AICAR (P<0.05) (Fig. 2A, D, E). Thus, the activated AMPK level during inflammation was, at least in part, responsible for the ERG response, and the visual function.

### Protective effect of AICAR on the rhodopsin level and OS length during inflammation

**Figure 3:**
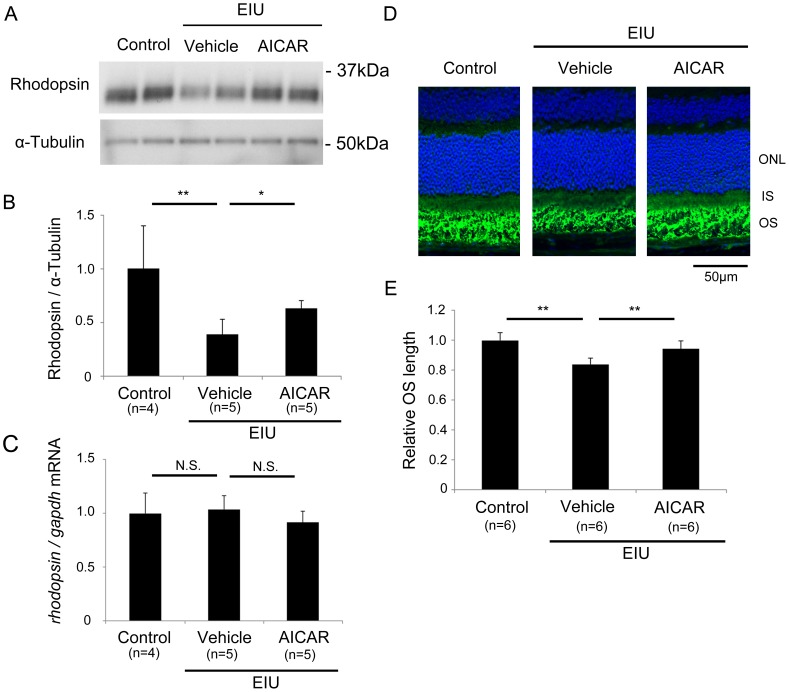
Protective effect of AICAR on the rhodopsin level and OS length during inflammation. (A, B) Immunoblot analysis. Rhodopsin protein in the retina was decreased during EIU, and this decrease was attenuated by AICAR, 24 hours after LPS injection. (C) Real-time PCR. rhodopsin mRNA was constant 24 hours after LPS injection with or without AICAR treatment. (D) The OS length was shortened during EIU, and this influence was suppressed by AICAR. (E) Relative OS length was measured in the mid-peripheral retina. *P<0.05. **P<0.01. Scale bar, 50 µm. (A–C) Control, n = 4; EIU with vehicle treatment, n = 5; EIU with AICAR treatment, n = 5. (D, E) All groups, n = 6. ONL, outer nuclear layer; IS, inner segment; OS, outer segment.

Since the ERG results showed that AICAR had a protective effect on photoreceptor cell function, we next analyzed the level of rhodopsin, a visual pigment that is indispensable for transducing light stimuli to neuronal electrical activity, in the retina 24 hours after LPS injection. Immunoblot analysis showed a reduction in the rhodopsin level during inflammation, and this reduction was attenuated by AICAR treatment (P<0.05) (Fig. 3A, B).

Because our previous data showed that rhodopsin reduction during inflammation is regulated at the post-transcriptional level [25], we analyzed rhodopsin mRNA level after AICAR treatment, to find that the level was not changed during inflammation with or without AICAR treatment (Fig. 3C). The data was confirmed by normalizing rhodopsin mRNA to β-actin and 36B4 (data not shown), as well as gapdh. Thus, the AICAR’s effect on rhodopsin protein preservation was through suppression of the post-transcriptional mechanism induced during inflammation.

We next measured the length of the photoreceptor OSs, where rhodopsin protein is delivered and concentrated at the plasma membrane. The OSs were shortened during inflammation, and this shortening was also suppressed by AICAR (P<0.01) (Fig. 3D, E).

Therefore, molecular and morphological changes in the photoreceptor cells during inflammation were both avoided by preserving AMPK activation by AICAR treatment.

### Suppressive effect of AICAR on NF-κB activation during inflammation

**Figure 4:**
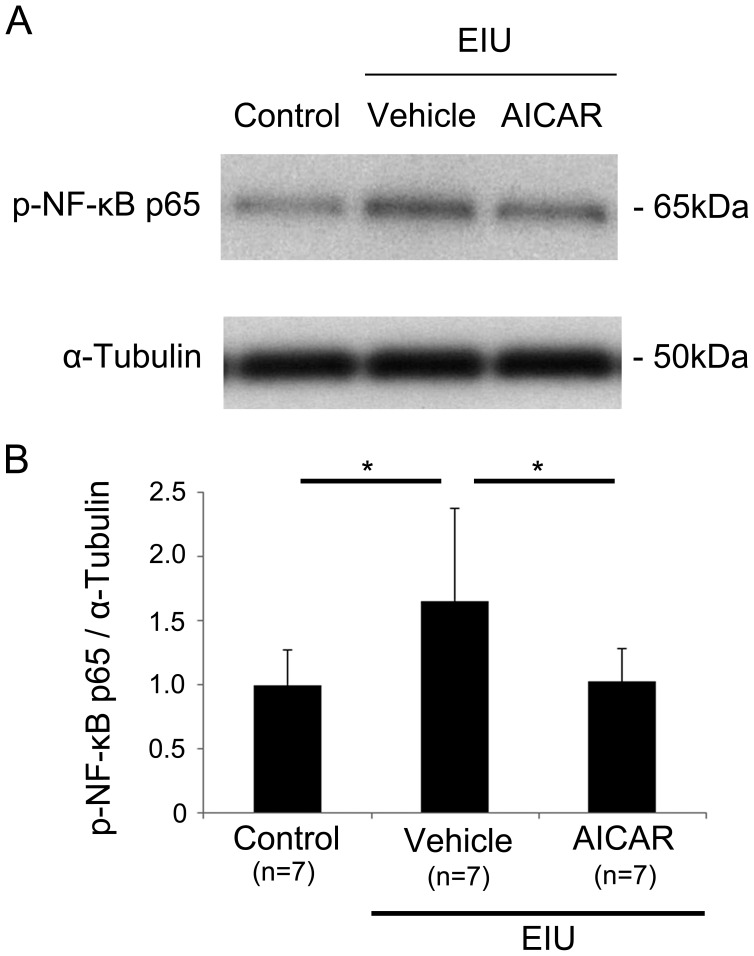
Suppressive effect of AICAR on NF-κB activation during inflammation. (A, B) Immunoblot analysis. The level of p-NF-κB p65 was increased in the retina 1.5 hours after LPS injection. AICAR significantly blocked the increase of the p-NF-κB p65 level during EIU. *P<0.05. All groups, n = 7. p-NF-κB p65, phosphorylated NF-κB p65.

Next, we measured the level of NF-κB, a transcription factor that can be activated during inflammation and has multiple downstream effectors. Immunoblot analysis showed that the level of phosphorylated p65, a component of activated NF-κB, was increased in the retina during retinal inflammation, by 1.5 hours after LPS injection, (P<0.05) (Fig. 4A, B). However, with AICAR treatment, the increased activation of NF-κB was suppressed in the inflamed retina.

### Preservation of the rhodopsin level by an inhibitor of NF-κB activation, DHMEQ, during inflammation

**Figure 5:**
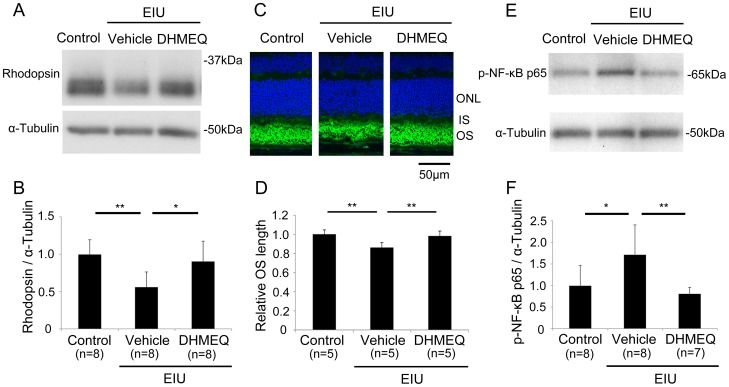
Preservation of rhodopsin level by an inhibitor of NF-κB activation, DHMEQ, during inflammation. (A, B) Immunoblot analysis. The rhodopsin level measured 24 hours after LPS injection was preserved by DHMEQ treatment. (C, D) The shortening of OS length during EIU was avoided by DHMEQ. Relative OS length was measured in the mid-peripheral retina. (E, F) Immunoblot analysis. The level of p-NF-κB p65 was decreased by DHMEQ in the retina 1.5 hours after LPS injection. *P<0.05. **P<0.01. Scale bar, 50 µm. (A, B) All groups, n = 8. (C, D) All groups, n = 5. (E, F) Control, n = 8; EIU with vehicle treatment, n = 8; EIU with AICAR treatment, n = 7. p-NF-κB p65, phosphorylated NF-κB p65.

We further analyzed the pathological role of activated NF-κB during retinal inflammation. For this purpose, we injected mice with DHMEQ, which suppresses the NF-κB’s translocation into the nucleus and DNA binding. Interestingly, DHMEQ prevented the reduction in the rhodopsin level during inflammation 24 hours after LPS injection (P<0.05) (Fig. 5A, B). To further analyze the biological effect of DHMEQ during inflammation, we measured OS length after DHMEQ treatment. Consistent with the rhodopsin level, OS shortening was avoided by DHMEQ (Fig. 5C, D). Moreover, to know the effect of DHMEQ which inhibits nuclear translocation of NF-κB, on the protein level of activated NF-κB, we performed immunoblot analysis to find that the level itself was also decreased by DHMEQ in the retina 1.5 hours after LPS injection (Fig. 5E, F).

Taken together, NF-κB activation in response to the reduction of activated AMPK level was, at least in part, responsible for the photoreceptor cell damage.

## Discussion

In this study, we demonstrated that activated AMPK in the neural retina was decreased during inflammation, but its level was preserved by AICAR administration (Fig. 1). The visual function demonstrated by ERG showed impaired photoreceptor cell function during inflammation, and this impairment was prevented by AICAR (Fig. 2). Consistent with these findings, the level of rhodopsin and the length of OSs in the photoreceptor cells were reduced during inflammation, and these changes were also prevented by AICAR without changing rhodopsin mRNA level (Fig. 3). In addition, by using the NF-κB inhibitor, DHMEQ, we showed that increased NF-κB activation in the retina during inflammation, which was suppressed by AICAR (Fig. 4), was responsible for reducing the rhodopsin level during inflammation (Fig. 5).

The activated AMPK level was reduced in the retina at the early phase of inflammation. This finding is consistent with the previous observation that pro-inflammatory stimuli, such as LPS administration, reduce the level of activated AMPK, whereas anti-inflammatory stimuli increase it, in both mouse and human macrophage cell lines [30], [31]. In addition, angiotensin II decreases the activated AMPK [32] in cardiomyocytes. Because pro-inflammatory signals such as IL-6 [23] and angiotensin II [21] are induced in the retina during inflammation, as we reported previously, the reduction of activated AMPK that we observed in this study may have been, at least partly, influenced by the pro-inflammatory cytokines produced in the retina during inflammation.

Moreover, immunohistochemical analysis showed the local change of the AMPK status in the neural retina, suggesting that the data in this study were, at least in part, related to AMPK activation in the retinal neurons including photoreceptor cells. The expression of AMPK is previously reported in the hippocampal neurons [33], consistent with our observation in terms of AMPK being activated in the neurons.

AICAR, which promoted AMPK activation during inflammation, had an obvious effect on the preservation of the a-wave in the ERG, indicating that it was protective for photoreceptor cell function. The amplitude of the a-wave correlated positively with the rhodopsin level and OS length of the photoreceptor cells. Causal relationships between the rhodopsin level and OS length, and between the rhodopsin level and photoreceptor function, have been established using homo- and hemizygotes of rhodopsin-knockout mice [34], [35]. Taking all of these findings together, we conclude that the decrease in rhodopsin during inflammation induced both the OS shortening and the photoreceptor dysfunction recorded by ERG. The absence of obvious photoreceptor cell death during retinal inflammation in contrast to rhodopsin knockout mice was confirmed previously [24]. This would be because the model has only one injection of LPS, thus, the pathological findings, including reduction of activated AMPK and increase in the NF-κB activation, are going to be recovered afterwards (Fig. S1 and S2). Therefore, rhodopsin level also recovers, later, which we have previously reported [25]. Reduction in the amplitude of b-wave, which reflects the subsequent neuronal activity to the photoreceptor cells, during inflammation was also avoided by AICAR, most likely because the photoreceptor function was protected by AICAR.

The reduction in the rhodopsin level during inflammation is reported to occur post-transcriptionally, through excessive protein degradation via the ubiquitin proteasome system (UPS) [18], [25]. Consistent with the previous study, we observed a constant level of rhodopsin mRNA in the retina during inflammation, with or without AICAR treatment, suggesting that AICAR did not increase rhodopsin production, but instead may have suppressed rhodopsin degradation during inflammation, although the possibility of translation suppression could have been involved during inflammation. Given that AMPK activation was observed in the photoreceptor cells, the regulation of rhodopsin protein by AMPK activation may, at least in part, occur in a cell-autonomous manner.

Tissue protection by AMPK activation is widely reported in a variety of systems. For example, the activation of AMPK inhibits angiotensin II-induced vascular smooth muscle cell proliferation which can cause atherogenesis [36] or cardiomyocyte hypertrophy [32]. The decrease in rhodopsin and the visual dysfunction we observed in the EIU model during inflammation are also downstream effects of angiotensin II type 1 receptor signaling [21], and AICAR-induced AMPK activation inhibited these influences in the present study. Thus, it would be interesting to examine the relationship between angiotensin II and AMPK in the future. Moreover, AICAR treatment, which could replace the requirement of exercise in treating metabolic diseases [37], succeeded to suppress retinal inflammation and protect visual function in this study, proposing the possible explanation involved in the relationship between exercise and the low risk of retinal inflammatory diseases, such as age-related macular degeneration [38] and diabetic retinopathy [39].

The activation of NF-κB is suppressed by its binding with IκB in the cytoplasm. In response to certain kinds of stimuli, including inflammatory stimuli, IκB is phosphorylated, and subsequently degraded. NF-κB is then translocated into the nucleus and acetylated to promote the transcriptional activity of multiple target genes, which include pro-inflammatory mediators. In vitro experiments showing a relationship between AMPK activation and NF-κB have been reported [40], [41]. In these reports, the IκB kinase (IKK) α/β activity and the decrease in IκB in response to pro-inflammatory cytokines were suppressed through AMPK activation by AICAR in bone marrow neutrophils [40] and in a primary culture of astrocytes and microglial cells [41]. Moreover, a relationship between AMPK and IκB phosphorylation, as well as a possible direct inactivation of NF-κB activity by AMPK, were shown using AMPK-deficient mouse embryonic fibroblasts (MEFs) [42]. These findings are consistent with our result that NF-κB was activated during inflammation when AMPK activation was reduced, but was suppressed after AICAR treatment, which induces AMPK activation.

Various pro-inflammatory molecules can be produced downstream of NF-κB, such as IL-6 [23], which can further activate NF-κB and exacerbate disease, suggesting that NF-κB can be further activated in response to NF-κB’s targets. In fact, we observed that phosphorylated p65 increased during inflammation, and this increase was suppressed by DHMEQ, suggesting that DHMEQ may have decreased NF-κB’s action both directly by inhibiting its nuclear localization, as originally assumed [27]–[29], and indirectly by suppressing the positive feedback loop of NF-κB activation during inflammation. When AMPK activation was sufficiently preserved by AICAR from the early phase of inflammation, this vicious cycle of NF-κB activation may have been efficiently blocked. By using DHMEQ, we showed that inhibiting NF-κB’s action helped protect photoreceptor cells from inflammatory damage by inhibiting the decrease in rhodopsin. Therefore, the protective effect of AICAR on rhodopsin expression in the photoreceptor cells was exerted by NF-κB. The level of rhodopsin protein after treated with AICAR was not as high as control, even though the experiment has been done with the most effective dose for AICAR which was optimized by measuring rhodopsin level after treatment in this model (data not shown), suggesting that other pathways might also contribute to the reduction of rhodopsin protein during inflammation. It is not surprising that multiple pathways are involved in the inflammatory response.

The underlying molecular mechanism by which NF-κB decreases the rhodopsin level is currently unclear. However, given that this reduction occurs at least partly through UPS-mediated excessive degradation [18], [25], NF-κB may promote UPS-related gene expression. Notably, the promoter regions of various genes of the UPS pathway contain potential binding sites for NF-κB [43]; therefore, the redox-sensitive transcription factor NF-κB might have facilitated UPS-dependent proteolysis in the retina during inflammation. Transgenic mice specifically overexpressing NF-κB in muscle show muscle atrophy due to UPS-mediated protein degradation, resulting from the overexpression of a muscle-specific E3 ligase, *MuRF1* [44]. In addition, the induction of *MuRF1* in diaphragm muscle during inflammation by NF-κB was shown using transgenic mice in which “muscle IκBα super repressor” (MISR) signaling is repressed in the muscle [45]. These previous reports support the possibility that NF-κB induced UPS-mediated protein degradation in the inflamed retina, although further studies are required.

In summary, the NF-κB activation and subsequent decrease in rhodopsin, together with the visual dysfunction induced by inflammation in the retina were prevented by AICAR, which preserved the level of activated AMPK. Therefore, the AMPK activation was proposed as an effective approach for neuroprotective therapy during inflammation through suppressing NF-κB activation.

## Supporting Information

Figure S1 **Time course of activated AMPK level in the retina after LPS injection measured by immunoblot analysis.** *P<0.05. All groups, n = 4.
(TIF)

Figure S2 **Time course of activated NF-κB level in the retina after LPS injection measured by immunoblot analysis to p-NF-κB p65.** *P<0.05. All groups, n = 4. p-NF-κB p65, phosphorylated NF-κB p65.
(TIF)
