# Supplementary figures and images for: AMPK-NF-κB Axis in the Photoreceptor Disorder during Retinal Inflammation
Source: PLoS One. 2014 Jul 21;9(7):e103013. doi: 10.1371/journal.pone.0103013 (PMC4105543; doi:10.1371/journal.pone.0103013)

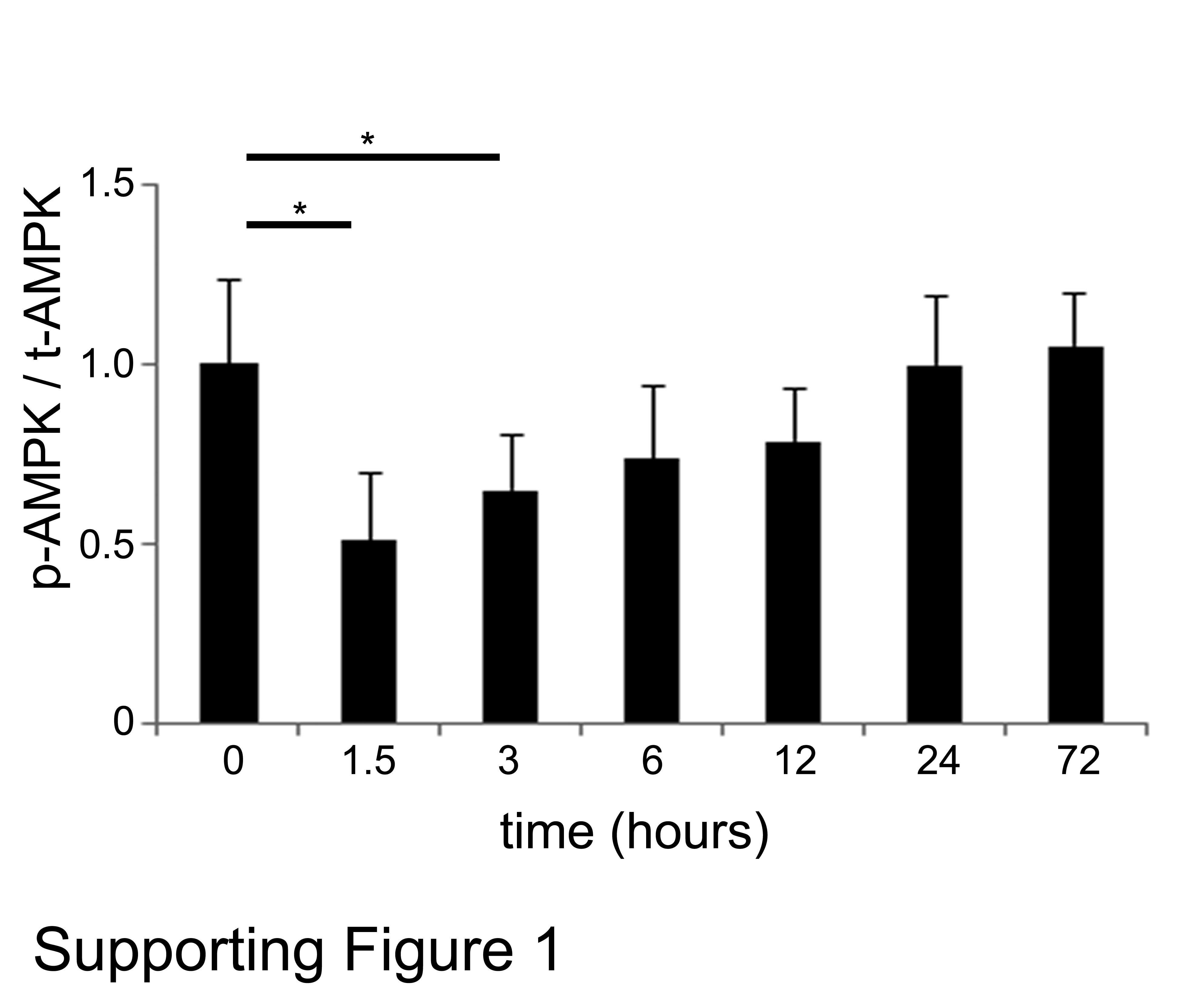

Supplement: Figure S1 — Time course of activated AMPK level in the retina after LPS injection measured by immunoblot analysis. *P<0.05. All groups, n = 4. (TIF) [file pone.0103013.s001.tif]

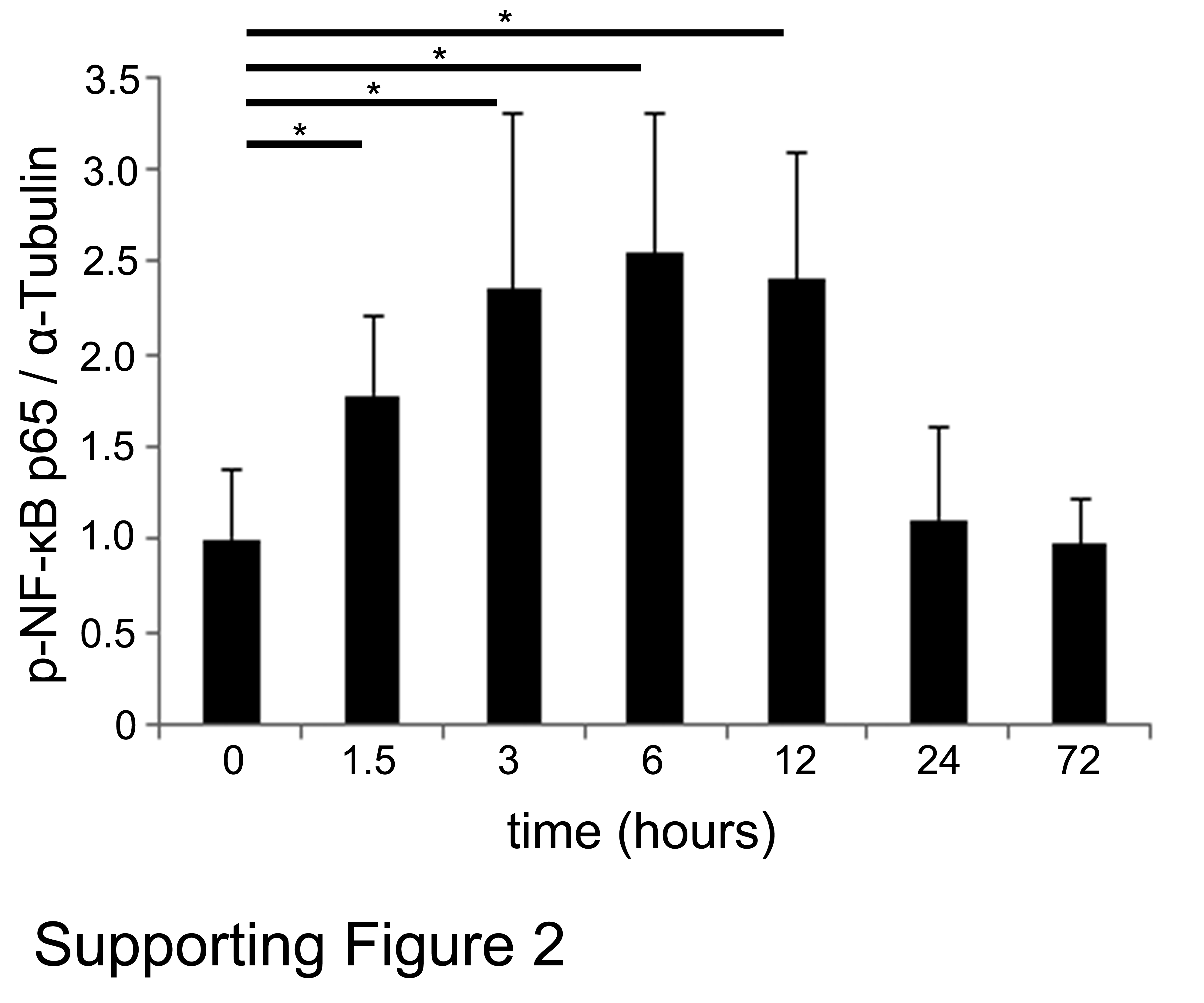

Supplement: Figure S2 — Time course of activated NF-κB level in the retina after LPS injection measured by immunoblot analysis to p-NF-κB p65. *P<0.05. All groups, n = 4. p-NF-κB p65, phosphorylated NF-κB p65. (TIF) [file pone.0103013.s002.tif]
